# Northern Ireland Food Safety Night

**DOI:** 10.3201/eid0911.030491

**Published:** 2003-11

**Authors:** John E. Moore

**Affiliations:** * Belfast City Hospital, Belfast, Northern Ireland

A meeting on food safety entitled “Food Safety: Is It All ‘“Pie-in-the-Sky?’“was held on June 19, 2003, in Belfast, Northern Ireland. Because Northern Ireland has a largely agrarian economy with a strong agricultural food sector, highlighting the importance of safe food production was timely. The meeting was organized by the Northern Ireland Microbiology Discussion Group (NIMDG) and was well attended by several local stakeholders in food safety, including representatives from hospital and public health microbiology laboratories, the Department of Agriculture and Rural Development of Northern Ireland, academia, and the food industry. The group heard presentations from Hugh Pennington, Department of Medical Microbiology, University of Aberdeen, Aberdeen, United Kingdom, and. Mike Kelly, Head of Food Safety and Environmental Health, British Airways.

Professor Pennington examined risk assessment in food safety, and discussed the approach to risk assessment of the oil and rail industries, and compared risk assessment between these industries and the food industry. He emphasized that, although science is important at addressing fundamental issues, an important emerging strategy is the translation of scientific findings into everyday practice to ensure that food safety is maintained. The attendees concluded that a greater interaction between microbiologists and psychologists should be encouraged, to add value to the science and ensure tangible benefits in reducing the incidence of foodborne illnesses. The group also learned that differences exist in the incidence of foodborne illnesses between Northern Ireland and Great Britain (England and Wales/Scotland), particularly with *Campylobacter* and *Salmonella* infections, and explored possible reasons for such differences, including climate, lack of consumption of unpasteurized milk, and the social custom of having foods “well done.”

Mr. Kelly described 42 documented cases of foodborne infections found in the literature that involved contaminated foods on aircraft and detailed, in practical terms, how the concept of a hazard analysis critical control point (HACP) has been used successfully by airline caterers to reduce such infections to a rare occurrence.

